# Calorie Restriction Effect of Heat-Processed Onion Extract (ONI) Using In Vitro and In Vivo Animal Models

**DOI:** 10.3390/ijms19030874

**Published:** 2018-03-15

**Authors:** Yu-Ri Kang, Hwang-Yong Choi, Jung-Yun Lee, Soo In Jang, Hanna Kang, Jung-Bae Oh, Hae-Dong Jang, Young-In Kwon

**Affiliations:** 1 Department of Food and Nutrition, Hannam University, Daejeon 34054, Korea; djsdnd12@nate.com (Y.-R.K.); kelolo123@naver.com (H.-Y.C.); seembeeks@hanmail.net (J.-Y.L.); sooin8042@nate.com (S.I.J.); hanna9506@daum.net (H.K.); 2 Institute of Functional Foods, KunpoongBio Co. Ltd., Jeju 63010, Korea; denisoh89@naver.com

**Keywords:** heat-process, onion, calorie restriction, Amadori rearrangement compounds, hyperglycemia

## Abstract

Onion (*Allium cepa* L.) is widely consumed as food or medicinal plant due to its well-defined health benefits. The antioxidant and antihyperlipidemic effects of onion and its extracts have been reported well. However, very limited information on anti-hyperglycemic effect is available in processed onion extracts. In our previous study, we reported that Amadori rearrangement compounds (ARCs) produced by heat-processing in Korean ginseng can reduce carbohydrate absorption by inhibiting intestinal carbohydrate hydrolyzing enzymes in both in vitro and in vivo animal models. To prove the enhancement of anti-hyperglycemic effect and ARCs content by heat-processing in onion extract, a correlation between the anti-hyperglycemic activity and the total content of ARCs of heat-processed onion extract (ONI) was investigated. ONI has a high content of ARCs and had high rat small intestinal sucrase inhibitory activity (0.34 ± 0.03 mg/mL, IC_50_) relevant for the potential management of postprandial hyperglycemia. The effect of ONI on the postprandial blood glucose increase was investigated in Sprague Dawley (SD) rats fed on sucrose or starch meals. The maximum blood glucose levels (C*max*) of heat-processed onion extract were significantly decreased by about 8.7% (from 188.60 ± 5.37 to 172.27 ± 3.96, *p* < 0.001) and 14.2% (from 204.04 ± 8.73 to 175.13 ± 14.09, *p* < 0.01) in sucrose and starch loading tests, respectively. These results indicate that ARCs in onion extract produced by heat-processing have anti-diabetic effect by suppressing carbohydrate absorption via inhibition of intestinal sucrase, thereby reducing the postprandial increase of blood glucose. Therefore, enhancement of ARCs in onion by heat-processing might be a good strategy for the development of the new product on the management of hyperglycemia.

## 1. Introduction

Hyperglycemia is a common symptom of diabetes with blood sugar levels higher than normal and is a condition in which an excessive amount of glucose circulates in the blood plasma. Chronic hyperglycemia causes various complications such as nephropathy, retinopathy, neuropathy, stroke, and cardiovascular diseases in type 2 diabetes mellitus (T2DM) patients [1]. Therefore, regulation of postprandial hyperglycemia is an important strategy used for treatment in T2DM patients. Dietary carbohydrates are broken down into monosaccharides by carbohydrate hydrolyzing enzymes such as α-amylase and α-glucosidases (sucrase, maltase, and glucoamylase) before their absorption [2]. Inhibition of these enzymes can suppress or retard the absorption of monosaccharides such as glucose, fructose, and galactose. Modulation of activities of these enzymes can prevent postprandial hyperglycemia and maintain normal blood glucose levels [3].

Recent studies report that intestinal α-glucosidase inhibitors from medicinal plants could be as effective as commercial drugs with lower side effect [4]. Phytochemicals present in medicinal plants have various health-promoting activities and have been shown to exert intestinal α-glucosidase inhibitory effects in vitro [5].

Herbs, onions, and beans that are important and traditional nutritive components have elevated polyphenol contents [1]. Plants belonging to the *Allium* species, such as onions and garlic, are used as foodstuffs, condiments, flavorings, and folk medicines [2]. Onion is rich in flavonoids such as quercetin and organosulfur compounds such as allyl propyl disulfide and S-methyl cysteine sulfoxide. It was reported that quercetin has antioxidant, anti-inflammatory, and antidiabetic effect [6,7,8]. Moreover, S-methyl cysteine sulfoxide has a lipid-lowering, antidiabetic, and antioxidant effects in alloxan diabetic rats [9,10]. The degree to which phytochemicals change during processing depends on the sensitivity of the phytochemical to modification or degradation, and length of exposure to a processing technique [11,12]. The content of quercetin in processed vegetables is dramatically decreased in the alkaline conditions, especially during heat-processing [10]. These results indicate that food processing conditions play an important role for food quality and functionality.

In addition, specific compounds, such as Amadori rearrangement compounds (ARCs) and Maillard reaction produts (MRPs), are produced by the heat-treatment of onions. It is reported that the production of MRPs and ARCs such as arginyl-fructose (AF) and fructosyl-lysine due to heat-treatment of onion results to the increase of antioxidant activity of onions [13,14,15].

During steaming and drying processes that are necessary for the production of Korean red ginseng or onion, certain components undergo non-enzymatic browning reaction, otherwise known as a Maillard reaction [16]. In the early stage of Maillard reaction, AF and arginyl-fructosyl-glucose (AFG) are formed through Amadori rearrangement of arginine with glucose or maltose in ginseng or onion, respectively [17]. In our previous study, we reported that arginyl-fructose (AF) and arginyl-fructosyl-glucose (AFG) produced by heat-processing in Korean ginseng can reduce carbohydrate absorption by inhibiting intestinal carbohydrate hydrolyzing enzymes in both in vitro and in vivo animal models [16,17]. The postprandial anti-hyperglycemia effect of AF from heat-processed Korean ginseng in db/db animal model and human clinical trials was also reported by our previous study [18,19].

However, very limited information is available about the enhancement of ARCs content in plant foods, except for red ginseng and subsequent enhanced antidiabetic activity. Therefore, to investigate the benefit of the heat-processing of onions for improving the antidiabetic activity and maximizing the production of ARCs, it is essential to measure the effect of heat-processing. Consequently, the purpose of this study was (i) to identify how heat processing affects ARC production in onion extracts and (ii) to evaluate the anti-hyperglycemic effect of heat-processed onion extracts such as heat-treated onion extract containing high ARCs (ONI_H) and heat-treated onion extract containing low ARCs (ONI_L) using in vitro alpha-glucosidase and sucrase inhibition assays as well as an in vivo animal model.

## 2. Results

### 2.1. Sample Preparation

**Figure 1:**
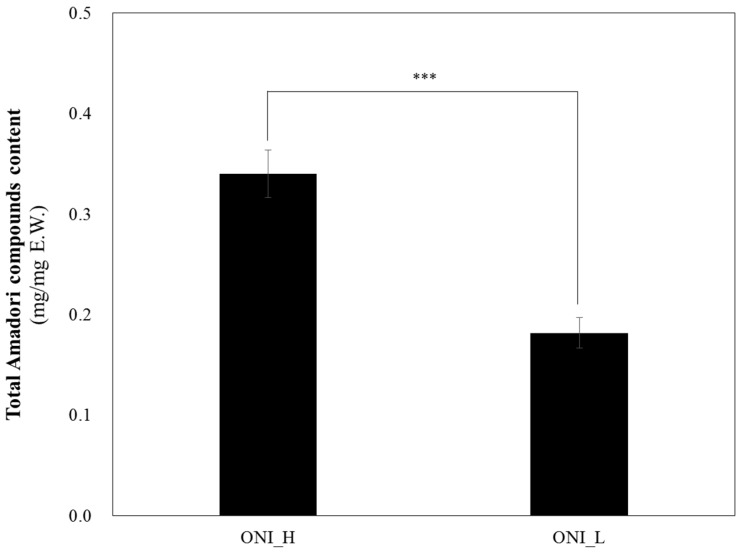
Total ARCs content of two different onion extracts (ONI_H and ONI_L). The results were expressed as mean ± S.D. Statistical significances were determined by Student’s *t*-test (*** *p* < 0.001).

As seen in Table 1 and Figure 1, two sample powders—which have a different contents of total ARCs, arginyl-fructose, glucose, fructose, and quercetin due to the different processing condition were prepared. Each sample was named by the content of ARCs, ONI_H (13.3% of AF and 33% of total ARCs), and ONI_L (5.55% of AF and 18% of total ARCs), respectively. In regards to ARC contents ONI_H and ONI_L treatments resulted to significantly higher contents of ARC when compared to quercetin (Table 1). These results indicate that reaction time for heating onions is an important factor for producing ARCs. Furthermore, we observed that ONI_H had higher ARC and quercetin contents, when compared to ONI_L (Table 1). Although both ARC and quercetin contents increased with heat treatment, since ARCs account for 38.02% of the total weight of the tested extract, we suspect that the observed bioactivities are due to ARCS, but we cannot exclude the possible synergistic effect of quercetin, which has a well-defined anti-hyperglycaemic effect.

### 2.2. α-Glucosidase Inhibitory Activity

The α-glucosidase inhibitors, which interfere with enzymatic action in the brush-border of the small intestine, could inhibit the liberation of d-glucose from oligosaccharides and disaccharides, resulting in reduced postprandial plasma glucose levels.

**Figure 2:**
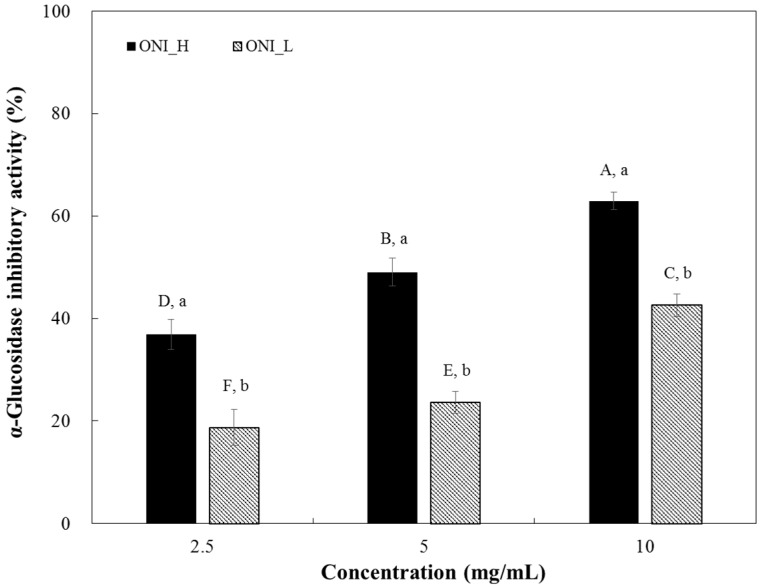
Dose dependent changes in rat small intestinal α-glucosidase inhibitory activities (% inhibition) of two different onion extracts (ONI_H and ONI_L). The results were expressed as mean ± S.D. with three independent experiments in triplicate. Different corresponding letters indicate significant differences at *p* < 0.05 by Duncan’s test. ^A–F^ The first letters in uppercase indicate significant differences among all samples and ^a–b^ the second letters in lowercase are different between ONI_H and ONI_L within the same concentration.

**Figure 3:**
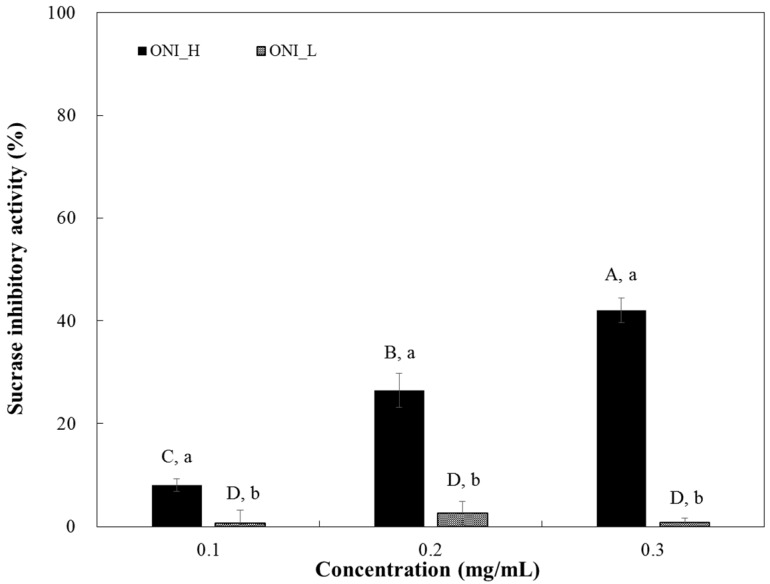
Dose dependent changes in rat small intestinal sucrase inhibitory activities (% inhibition) of two different onion extracts (ONI_H and ONI_L). The results were expressed as mean ± S.D. with three independent experiments in triplicate. Different corresponding letters indicate significant differences at *p* < 0.05 by Duncan’s test. ^A–D^ The first letters in uppercase indicate significant differences among all samples and ^a–b^ the second letters in lowercase are different between ONI_H and ONI_L within same concentration.

α-Glucosidase inhibitory activities of ONI_H and ONI_L are shown in Figure 2. As expected, ONI_H (high ARCs content sample), showed higher α-glucosidase inhibitory activity (5.87 mg/mL of IC_50_) (Table 2) than that of ONI_L (>12.59 mg/mL of IC_50_) (Table 2). In terms of sucrase inhibition, ONI_H inhibits sucrase activity in a dose-dependent manner, but ONI_L showed almost no inhibition at all tested doses (Figure 3). The half-maximal inhibitory concentration (IC_50_) of ONI_H was 0.34 mg/mL.

As seen in Figure 2 and Figure 3, ONI_H which has a higher content of AF and ARC than ONI_L showed high α-glucosidase and sucrase inhibitory activities. Interestingly, inhibitory activity of both ONI_H and ONI_L against sucrase was higher than that of α-glucosidase. This result may due to the enzyme inhibition specificity of AF, a major ingredient in both extracts. According to a previous report by Ha et al. (2011), AF yielded to superior inhibitory effect against sucrase (IC_50_, 6.40 mM) [17] suggesting possible bioactive components were present in the heat-processed onions.

Our observations suggest that the increased content of AF or ARCs is critical for enzyme inhibitory activity of heat-processed onion extract. These results indicate that ARCs produced by heat treatment plays an important role in the enhancement of functionality of onion-based products.

### 2.3. Blood Glucose Lowering Effect of Onion Extracts In Vivo

**Figure 4:**
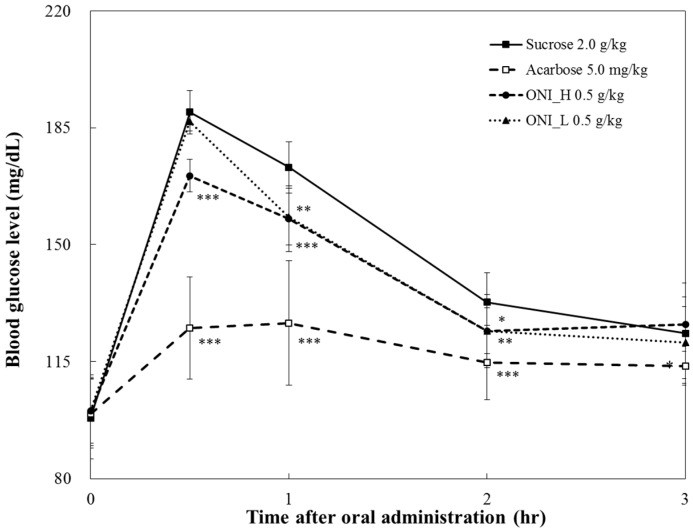
Comparison of postprandial blood glucose-lowering effects of ONI_H and ONI_L in sucrose loading test. After fasting for 24 h, five-week-old, male SD rats were orally administered sucrose solution (2.0 g/kg) with or without samples (onion extracts). The results were expressed as mean ± S.D. Statistical significances were determined by Student’s *t*-test (* *p* < 0.05; ** *p* < 0.01; and *** *p* < 0.001).

ONI_H and ONI_L showed significant inhibition against α-glucosidases especially for sucrase, which is a membrane-bound enzyme at the epithelia of the small intestine and a key enzyme of sucrose digestion (Table 2). Inhibition of sucrase may lead to a delayed and reduced rise in postprandial blood glucose levels. To confirm the in vitro sucrase inhibitory activity of samples, the in vivo blood glucose reducing effects of ONI_H and ONI_L were evaluated with SD rats and the results are illustrated in Figure 4.

In the ONI_L-treated group with sucrose, the blood glucose level was 186.90 ± 2.92 mg/dL at 30 min after administration and not significantly different to the sucrose control group (189.67 ± 6.50 mg/dL) (Figure 4). On the other hand, ONI_H-treated group (170.63 ± 4.87 mg/dL, *p* < 0.001) suppresses the rising of plasma glucose level by 19.04 mg/dL compared to the sucrose control group at 30 min after administration.

**Figure 5:**
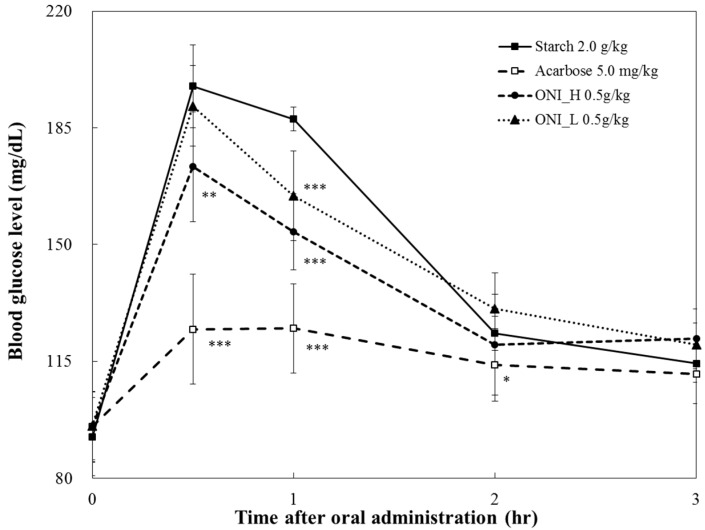
Comparison of postprandial blood glucose-lowering effects of pyridoxine, pyridoxal, and pyridoxamine in starch loading test. After fasting for 24 h, five-week-old, male SD rats were orally administered starch solution (2.0 g/kg) with or without samples (onion extracts). The results were expressed as mean ± S.D. Statistical significances were determined by Student’s *t*-test (* *p* < 0.05; ** *p* < 0.01; and *** *p* < 0.001).

In terms of starch loading test, blood glucose level in the ONI_H-treated group was 173.33 ± 16.41 mg/dL (*p* < 0.01) at 30 min after administration (Figure 5). This is lower than the ONI_L-treated group (191.60 ± 12.08 mg/dL) and the starch group (197.50 ± 12.42 mg/dL) by 18.27 mg/dL and 24.17 mg/dL respectively. At 1 h after administration, the blood glucose level of ONI_H-treated group (153.82 ± 11.44 mg/dL, *p* < 0.001) was lower than both the ONI_L-treated group (164.62 ± 13.43 mg/dL) and starch group (187.67 ± 3.51 mg/dL).

### 2.4. Pharmacodynamics Parameters

Pharmacodynamic (PD) parameters of the sucrose and starch loading tests are shown in Table 3. In terms of T*max*, there is no significant difference among sucrose and both ONI_H and ONI_L-treated groups. In contrast, ONI_H-treated groups resulted significantly reduced C*max* and AUC*t*, however this reduction was less effective than the acarbose-treated group. Specifically for C*max*, the maximum blood glucose levels (C*max*) of ONI_H administration group decreased by about 8.6% (from 188.60 ± 5.37 to 172.27 ± 3.96) and 14.3% (from 204.04 ± 8.73 to 175.13 ± 14.09) in sucrose and starch loading tests, respectively, when compared to control in pharmacodynamics study.

Our findings suggest health beneficial effect of ONI_H against high blood glucose levels, following sucrose and starch administration. Based on the above observations, it is expected that the resulting reduction of glucose levels could be due to the inhibitory effect of ONI_H against carbohydrate hydrolyzing enzymes.

## 3. Discussion

It is reported that the production of Maillard reaction products (MRPs) and Amodori rearrangement compounds (ARCs) such as arginyl-fructose and fructosyl-lysine due to heat-treatment of onion positively affects the antioxidant activity of onion [11,12,13]. ARCs such as arginyl-fructose (AF) and arginyl-fructosyl-glucose (AFG) in red ginseng possess antioxidant and antidiabetic activity [14,15]. However, very limited information is available about the enhancement of ARCs content in various plant foods extracts, except for red ginseng [20,21,22].

In this study, the optimum condition for improving the antidiabetic activity and maximizing the production of ARCs in onion extract was investigated. ARCs production was positively proportional to the reaction temperature and controlled by the amount of arginine and food-grade acid additions. ARCs content was also correlated with inhibitory activity against α-glucosidase and sucrase, which are membrane-bound enzymes at the epithelia of the small intestine and key enzymes of carbohydrate digestion.

Furthermore, to confirm the in vitro α-glucosidase and sucrase inhibitory activities of samples, the in vivo blood glucose reducing effects of onion extract with high ARCs content (ONI_H) and low ARCs content (ONI_L) were evaluated with SD rats and the results were similar with in vitro data. The postprandial blood glucose levels of the ONI_H-treated group were decreased more effectively than the ONI_L-treated group in sucrose and starch loading tests.

These results suggest that ARCs—such as AF—in heat-processed onions could be the reason for the resulting reduction in postprandial blood glucose levels, as previously described by Ha et al. [15]. Also, it is reported that ARCs from ginseng could be great antioxidant compounds [14]. Even though the quercetin contents were significantly lower when compared to ARC contents, we cannot exclude the possible synergistic effect of ARCs and quercetin, since the anti-diabetic effect of quercetin is well-defined. Our findings suggest that the observed postprandial glucose reduction effect of ONI_H correlate to ARCs and quercetin contents and therefore can have an additional health-benefits, such as the reduction of oxidation-induced complication of diabetes.

These results indicate that heat-processing of onion supplemented with arginine resulted in the formation of ARCs which could be effectively designed as complementary therapies for postprandial hyperglycemia linked to type 2 diabetes prevention.

## 4. Materials and Methods

### 4.1. Materials

Korean onion (*Allium cepa* L.) was purchased from a local market in Jeju, Korea. Rat intestinal acetone powders of α-glucosidase (EC 3.2.1.20) and nitro blue tetrazolium (NBT) were purchased from Sigma-Aldrich Co. (St. Louis, MO, USA). Sodium carbonate was purchased from DUKSAN Pure Chemicals Co. (Ansan, Kyounggi-Do, Korea). Unless noted, all chemicals were purchased from Sigma-Aldrich Co. (St. Louis, MO, USA).

### 4.2. Sample Preparation

In order to produce a high mass of heat-processed onion extract, the scale-up process with 500 kg onions was performed, using the facilities in KunpoongBio Co., Ltd. (Jeju city, Jeju-Do, Korea). The onions, whole without peeling, were crushed and enzymatically digested with 0.5% Celluclast + Pectinex (Novozyme Korea Ltd., Seoul, Korea) mix for 2 h at 50 °C, and then the enzyme was inactivated for 20 min at 100 °C and the filtered using a sieve (10 mesh). The filtrate sieved (10 mesh) was concentrated at 60 °C using vacuum evaporator. Citric acid and arginine were used as a pH regulator. It was then heated for 3 or 5 h at 90 °C. Finally, the two reactants such as 3 h (ONI_L) and 5 h (ONI_H) reactants were spray-dried, respectively. After all this process, total ARCs, arginyl-fructose, residue glucose, fructose, and quercetin contents of the two samples were measured. Finally, dextrin—an extender—was added and spray dried. Samples were stored at −20 °C until analysis.

### 4.3. Total Amadori Compounds Analysis

Fructosamine measurement method was modified and used as an experimental method [23]. Sodium carbonate was prepared as 0.1 M, pH 10.3 and 0.57 mM of NBT dissolved in 0.1 M sodium carbonate was prepared. The sample solution (50 μL) and NBT solution (150 μL) was added to each well and incubated at 37 °C. The absorbance was measured at 540 nm at 10 and 20 min time points. The concentration of total ARCs was calculated compared to fructosyl-arginine as a standard.

### 4.4. α-Glucosidase Inhibition Assay

Rat intestinal α-glucosidase assay referred to the method of Kwon et al. [3] with a slight modification. A total of 1 g of rat-intestinal acetone powder was suspended in 3 mL of 0.1 M sodium phosphate buffer (pH 6.9), and the suspension was sonicated 12 times for 30 s at 4 °C. After centrifugation (10,000× *g*, 30 min, 4 °C), the resulting supernatant was used for the assay. Sample solution (50 μL) and 0.1 M sodium phosphate buffer (pH 6.9, 100 μL) containing glucosidase solution (1.0 U/mL) was incubated at 37 °C for 10 min. After pre-incubation, 5 mM p-nitrophenyl-α-d-glucopyranoside solution (50 μL) in 0.1 M sodium phosphate buffer (pH 6.9) was added to each well at timed intervals. The reaction mixtures were incubated at 37 °C for 30 min. After incubation, absorbance was read at 405 nm and compared to a control which had 50 μL of buffer solution in place of the extract by micro-plate reader (SUNRISE; Tecan Trading AG, Salzburg, Austria). The α-glucosidase inhibitory activity was expressed as inhibition % and was calculated as follows:

$$\mathrm{Inhibition}\;(\%)=\left(\left[\frac{\Delta A_{405}^{\mathrm{Control}}-\Delta A_{405}^{\mathrm{Extract}}}{\left[\Delta A_{405}^{\mathrm{Control}}\right]}\right]\right)\times 100$$

### 4.5. Sugar Loading Test

Effect on hyperglycemia induced by carbohydrate loads in Sprague Dawley (SD) rats was determined by the inhibitory action of processed onion extract and acarbose on postprandial hyperglycemia [17]. All animal procedures were approved by Institutional Animal Care and Use Committee (IACUC) of the Hannam University (Approval number: HNU2017-003, 14/03/2017). Five-week-old male SD rats were purchased from Raon Bio Co. (Yongin, Kyounggi-Do, Korea) and fed a solid diet (Samyang Diet Co., Seoul, Korea) for one week. The rats were housed in a ventilated room at 25 ± 2 °C with 50 ± 7% relative humidity and under an alternating 12 h light/dark cycle. After 6 groups of 5 male SD rats (180–200 g) fasted for 24 h, 2.0 g/kg of sucrose were orally administrated concurrently with 0–500 mg/kg or onion extracts or acarbose. The blood samples were then taken from the tail after administration and blood glucose levels were measured at 0, 0.5, 1, 2, and 3 h. The glucose level in blood was determined by glucose oxidase method and compared with that of the control group, which had not taken the inhibitors. The parameters for blood glucose levels were calculated using PKsolver. Maximum observed peak blood glucose level (C*max*) and the time at which it is observed (T*max*) were determined based on the observed data. The area under the blood glucose–time curve up to the last sampled time-point (AUC*t*) was estimated by the trapezoidal rule.

### 4.6. Statistical Analysis

All data are presented as mean ± S.D. Statistical analyses were carried out using the statistical package SPSS 11 (Statistical Package for Social Science 11, SPSS Inc., Chicago, IL, USA) program and significance of each group was verified with the analysis of one-way ANOVA followed by Duncan’s test of *p* < 0.05. In addition, statistical significances in animal study were determined by Student’s *t*-test (* *p* < 0.05; ** *p* < 0.01; and *** *p* < 0.001).

## 5. Conclusions

Hyperglycemia has been identified as a major risk factor for cardiovascular complications linked to T2DM, and thereby known as an effective therapeutic target in the treatment of T2DM. Our data presented in this study strongly suggest that heat-processed onion extract can be a significant source of AF, a major bioactive ARCs in ONI, and phenolic compounds that exert postprandial blood glucose-lowering and antioxidant effects, respectively. Taking into consideration that AF is present in a wide variety of heat-processed food products, knowledge of this additional health benefit of heat-processed foods can assist in the development of efficacious anti-hyperglycemia supplements and give rationale for further clinical study.

Although further work is still needed to optimize the condition for production of anti-hyperglycemic components in the ONI and evaluate pharmacological effect in human clinical trial, our in vitro and in vivo data offer a biochemical rationale to support further clinical studies. These results also provide the basis for developing valuable alternatives to synthetic drugs to manage glycemic control from heat-processed food products in food industry.

## Figures and Tables

**Table 1:** The composition of two different onion extracts (ONI_H and ONI_L).

Samples	pH	Brix	AF (%)	Arg (%)	Glu (%)	Fru (%)	Que (ug/g)	Total ^1^ (%)
ONI_H	6.97	30.1	13.3	8.56	6.08	10.08	200	38.02
ONI_L	7.59	28.3	5.55	9.46	6.86	6.99	103	28.90

^1^ Total: AF + Arg + Glu + Fru; AF: arginyl-fructose, Arg: arginine, Glu: glucose, Fru: fructose, Que: quercetin.

**Table 2:** The half-maximal inhibitory concentration (IC_50_) values for rat intestinal α-glucosidase and sucrase by two different onion extracts (ONI_H and ONI_L).

IC_50_ (mg/mL)		
Enzymes	ONI_H	ONI_L
Sucrase	0.34 ± 0.03	ND ^1^
α-glucosidase	5.87 ± 0.60	>12.59 ± 0.27

^1^ ND—Not determined.

**Table 3:** Changes in pharmacodynamic (PD) parameters of control and after administration of ONI_H, ONI_L, and acarbose with sucrose or starch ingestions

Groups	PD Parameters		
	C*max* (mg/dL)	T*max* (h)	AUC*t* (h∙mg/dL)
Sucrose 2.0 g/kg	188.60 ± 5.37 ^a^	0.50 ± 0.00 ^b^	442.22 ± 18.45 ^a^
Acarbose 5.0 mg/kg	129.47 ± 15.84 ^c^	1.10 ± 0.55 ^a^	353.65 ± 34.41 ^b^
ONI_H 0.5 g/kg	172.27 ± 3.96 ^b^	0.50 ± 0.00 ^b^	418.11 ± 13.83 ^a^
ONI_L 0.5 g/kg	187.00 ± 1.90 ^a^	0.50 ± 0.00 ^b^	422.33 ± 14.38 ^a^
Starch 2.0 g/kg	204.04 ± 8.73 ^a^	0.75 ± 0.29 ^a^	451.90 ± 3.94 ^a^
Acarbose 5.0 mg/kg	133.43 ± 10.28 ^c^	1.00 ± 0.61 ^a^	350.48 ± 19.40 ^c^
ONI_H 0.5 g/kg	175.13 ± 14.09 ^b^	0.50 ± 0.00 ^a^	406.69 ± 22.62 ^b^
ONI_L 0.5 g/kg	193.77 ± 11.48 ^a^	0.50 ± 0.00 ^a^	434.95 ± 19.47 ^a^

The results were expressed as mean ± S.D. ^a–c^ Different letters indicate statistically significant differences between groups one-way ANOVA followed by Duncan’s test of *p* < 0.05.

## Abbreviations

ARCs	Amadori rearrangement compounds
SD rat	Sprague Dawley rat
MRPs	Maillard reaction products
AF	Arginyl-fructose
AFG	Arginyl-fructosyl-glucose
ONI_H	Heat-treated onion extract containing high ARCs
ONI_L	Heat-treated onion extract containing low ARCs
